# Impacts of Calculation Grid Spacing and Statistical Uncertainty of Monte Carlo Algorithm on Stereotactic Radiosurgery Planning With Volumetric-Modulated Arcs for Single Brain Metastases Using the Monaco® System

**DOI:** 10.7759/cureus.76325

**Published:** 2024-12-24

**Authors:** Kazuhiro Ohtakara, Kojiro Suzuki

**Affiliations:** 1 Department of Radiation Oncology, Kainan Hospital Aichi Prefectural Welfare Federation of Agricultural Cooperatives, Yatomi, JPN; 2 Department of Radiology, Aichi Medical University, Nagakute, JPN

**Keywords:** brain metastases, calculation grid size, concentric lamellarity, dose conformity, dose gradient, dose inhomogeneity, statistical uncertainty, stereotactic radiosurgery, volumetric-modulated arc therapy, x-ray voxel monte carlo

## Abstract

Purpose

In linac-based stereotactic radiosurgery (SRS) utilizing a multileaf collimator (MLC) for brain metastases (BMs), a volumetric-modulated arc (VMA) technique is indispensable for generating a suitable dose distribution with efficient planning and delivery. However, the optimal calculation grid spacing (GS) and statistical uncertainty (SU) of the Monte Carlo algorithm for VMA optimization have yet to be determined. This planning study aimed to examine the impacts of GS and GU settings on VMA-based SRS planning and to find the optimal combination for templating.

Materials and methods

Thirty clinical BMs with a gross tumor volume (GTV) of 0.08-48.09 cc (median 9.81 cc) were included. The treatment platform included a 5-mm leaf-width MLC Agility^®^ (Elekta AB, Stockholm, Sweden) and a planning system Monaco^®^ (Elekta AB). The prescribed dose was uniformly assigned to the GTV *D*_V-0.01 cc_, the minimum dose of GTV minus 0.01 cc, i.e., *D*_>95%_ for GTV >0.20 cc or to the GTV *D*_95%_ for GTV ≤0.20 cc, to minimize the uncovered GTV to the equivalent of a 3 mm diameter lesion. Five combinations of GS and SU per plan were examined for 12 selected GTVs (median 17.41 cc): GS of 2 mm and SU of 3% (G2U3), 2 mm and 2% (G2U2), 2 mm and 1% (G2U1), 1 mm and 2% (G1U2), and 1 mm and 1% (G1U1). Otherwise, the same arc arrangement and optimization method were uniformly used to prioritize the GTV dose conformity and the steepness of the dose gradient outside the GTV without dose constraints inside the GTV boundary. Further comparisons were conducted using 30 GTVs between the two groups with the highest plan quality.

Results

The G2U3 and G2U2 resulted in the equivalent total calculation time (tCT) and exactly the same plan quality. The overall plan quality was significantly superior in the G1U2 and G1U1 than in the G2U1 and G2U2, although the tCT was significantly longer in the G1U1 and G1U2 than in the G2U1 and G2U2. In the comparison of the G1U2 and G1U1, the concentric lamellarity of dose gradients 2 mm outside and 2-4 mm inside the GTV boundary was significantly superior in the G1U1 than in the G1U2, while there was no significant difference in the other parameters. The tCT tended to be longer in the G1U1 than in the G1U2.

Conclusions

The initial settings of GS and SU have significant impacts on the plan quality and tCT. The settings with GS of 1 mm and SU of 1% per plan are recommended to create the most suitable dose distribution for single BMs, especially for irregularly shaped and/or large lesions, although the tCT is long. In addition to common evaluation metrics, the coverage values of 2 mm outside and 2-4 mm inside the GTV surface by the *D*_eIIV_, the minimum dose to cover the irradiated isodose volume equivalent to each target volume, are valuable for in-depth plan comparison.

## Introduction

Stereotactic radiosurgery (SRS) is the least invasive, effective, and safe local therapeutic avenue for brain metastases (BMs) if properly designed, planned, and implemented [1]. Multi-fraction SRS enhances efficacy and safety by using flexible and appropriate dose fractionation according to the tumor volume, shape, localization, and proximity [1-3]. A volumetric-modulated arcs (VMA) technique is indispensable for generating a suitable dose distribution for linac-based SRS using a multileaf collimator (MLC) [4]. A conventional dynamic conformal arcs (DCA) technique is insufficient for ensuring appropriate target dose conformity, especially for non-spherical irregular lesions, so it is frequently necessary to leverage a modified target volume (TV) as a surrogate for MLC adaptation [5-7]. Even after much time spent on optimization through trial and error, DCA with a 2.5-mm leaf-width MLC is nowhere near as good as properly optimized VMA with a 5-mm leaf-width MLC, even in terms of TV dose conformity [8,9].

SRS for BMs is now performed using a variety of devices and irradiation techniques. There are substantial differences in target definition, dose distribution, and prescription methods among facilities, making comparisons of treatment contents and clinical results even more difficult [10,11]. Monaco^®^ (Elekta AB, Stockholm, Sweden) is one of the commonly used planning systems for VMA optimization [4,9]. There is a high degree of freedom in the VMA optimization method, i.e., in selecting and setting the CFs in Monaco^®^ [4,12,13]. At our facility, we prescribe a sufficient dose to the GTV boundary based on optimization that prioritizes the steepness of the dose gradient outside the GTV boundary and confirms that an appropriate dose attenuation margin outside the GTV is secured [9,14,15]. Monaco^®^ can provide a dose distribution suitable for SRS of BMs through simple optimization with just three physical cost functions (CFs) applying only to a gross tumor volume (GTV) itself and the body contour [9,16]. In addition to the excellent GTV dose conformity, another dosimetric advantage of VMA is the concentrically layered steep dose gradients outside and inside the GTV boundary [15,17].

The VMA optimization and dose calculation with Monaco^®^ is based on the X-ray voxel Monte Carlo algorithm (XVMC) [4,9]. The VMA planning with Monaco^®^ requires the selection of calculation grid spacing (GS) and statistical uncertainty (SU) settings for XVMC algorithms [18]. Limited previous studies examined the dosimetric effects of GS and SU settings on plan quality for VMAT planning [18-22]. However, the optimal GS and SU for VMA-based SRS of BMs have remained to be determined [19]. At our facility, from 2021 onwards, VMA-based SRS planning has been optimized with the GS 2 mm and SU 3% followed by the GS 1 mm for the final dose calculation to start the irradiation as soon as possible after image acquisition, preferably within 24 hours [9,23]. The initial settings of the GS and SU with smaller values may improve the plan quality.

This study was conducted to examine the impacts of GS and SU settings on planning and dosimetric parameters and to find the optimal values for templating in VMA-based SRS for single BMs.

This study was approved by the Clinical Research Review Board of Kainan Hospital Aichi Prefectural Welfare Federation of Agricultural Cooperatives (20240830-01).

## Materials and methods

This was a comparative planning study for the clinical scenarios of single BMs. Thirty lesions were extracted from 27 cases for which multi-fraction SRS was performed previously in our facility, to include a variety of volumes, localizations, and shapes. Each of the 30 lesions was treated as a single brain metastasis. Each GTV was defined and contoured using dedicated software, MIM Maestro^®^ version 7.1.3 (MIM Software Inc., Cleveland, OH, USA), as described previously [14,24]. The GTV ranged from 0.08 cc to 48.09 cc (median value: 9.81 cc; interquartile range [IQR]: 4.38, 24.31 cc).

The treatment platform consisted of a 160-leaf and 5-mm leaf-width MLC Agility^®^ (Elekta AB, Stockholm, Sweden) equipped with a linac Infinity^®^ (Elekta AB, Stockholm, Sweden) that provides a flattening filter-free mode of a 6 MV X-ray beam with the maximum dose rate of 1400 monitor units per minute [4,25]. The planning system used was Monaco^®^ version 5.51.10 (Elekta AB, Stockholm, Sweden) [4,12,13,16].

Each irradiation isocenter was set at the GTV center. Three arcs were uniformly arranged for each GTV, consisting of one coplanar arc with 360º rotation and the collimator angle of 0º and two NCAs with each 180º rotation, the collimator angles of 45º and 90º, and the couch rotations of 60º clockwise and counterclockwise, respectively, which were allocated to evenly divide the cranial hemisphere [26]. The increment (Inc) mechanical limiting parameter that controls the number of generated sectors was uniformly set to 20º per arc.

The VMA optimization was performed in the Pareto mode with priority given to maximizing the steepness of the dose gradient outside the GTV surface [16]. Three physical CFs were uniformly applied to each GTV and the patient’s head contour as described in Table 1.

**Table 1:** Structures and the selection and setting of cost functions. *The option of the multicriterial optimization is to continue to drive normal tissue sparing while maintaining the target dose. **In this setting, the cost function works for the area more than 2 mm outside the GTV boundary. GTV: gross tumor volume; PD: prescribed dose; *D*_V-0.01 cc_: the minimum dose to cover a target volume (TV) minus 0.01 cc (*D*_>95%_ for TV >0.20 cc, *D*_95%_ for TV ≤0.20 cc); RMS: root mean square.

Structure	Cost function	Parameter setting	
		Required	Optional
GTV	Target Penalty	Prescription (Gy): PD (e.g. 43.000 Gy); Minimum Volume (%): 95.00-99.98% (*D*_V-0.01 cc_)	Surface Margin +
Body contour (Patient)	Conformality	Relative Isoconstraint: 0.01	Margin Around Target: 8 cm; Multicriterial*: +
	Quadratic Overdose	Maximum Dose (Gy): PD (e.g. 43.000 Gy); RMS Dose Excess (Gy): 0.020	Multicriterial*: +; Shrink Structures**: GTV; Include +: Margin (cm) 0.20

The same prescribed dose was uniformly assigned to the GTV *D*_V-0.01 cc_, the minimum dose to cover a GTV minus 0.01 cc (*D*_>95%_ for GTV >0.20 cc and *D*_95%_ for GTV ≤0.20 cc), to minimize the uncovered GTV to the equivalent of a 3 mm diameter lesion, based on a previous study [14]. The GTV coverage values by the *D*_V-0.01 cc_ consequently ranged from 95.00% to 99.98% (median value: 99.90; interquartile range [IQR]: 99.77, 99.96).

A segment shape optimization (SSO) option with high-precision leaf positions of the value of 20 was included in the sequencing parameters for VMA [16]. Other parameters were uniformly set as the maximum control points of 1024 per arc, the minimum segment width of 0.5 cm, and the medium fluence smoothing, similar to previous studies [14-16]. The dose deposition of the XVMC algorithm was set to medium. Regarding the calculation of GS and SU per calculation for XVMC, five different combinations were compared using 12 GTVs as described in Table 2.

**Table 2:** Five combinations of calculation grid spacing and statistical uncertainties of the Monte Carlo algorithm. G: grid spacing (GS); U: statistical uncertainty (SU); GXUY: GS is X mm and SU is Y%.

Group abbreviation	G2U3	G2U2	G2U1	G1U2	G1U1
Grid spacing (mm)	2	2	2	1	1
Statistical uncertainty (%)	3	2	1	2	1

The 12 GTVs ranging from 0.08 cc to 48.09 cc (median value: 17.41 cc; IQR: 6.16, 32.06 cc) were selected from the 30 GTVs. In the grid spacing of 0.2 cm (G2U3, G2U2, and G2U1), the grid spacing was changed to 0.1 cm after the optimization, and the final dose calculation was performed. After the final dose calculation, each GTV coverage with the prescribed dose was rescaled according to each coverage value (≥95%) corresponding to the GTV *D*_V-0.01 cc_ [14,16]. A change in the GTV dose after rescaling of the GTV coverage by the prescribed dose was recorded to the third decimal place as the rescaling ratio, which was displayed as “Dose rescaled by a ratio of X” on Monaco^®^ [16].

The total calculation time (tCT) was defined as the time from the optimization initiation to the final control point completion for the GS of 0.1 cm or to the recalculation completion for the GS of 0.2 cm [19]. The tCT was determined from the optimization console on Monaco^®^ [19]. 

For dosimetric evaluation, isotropic margins of 2 mm, -2 mm, and -4 mm were added to each GTV boundary using MIM Maestro^®^ to generate the GTV + 2 mm, GTV - 2 mm, and GTV - 4 mm structures, respectively [15,17]. The GTV - 2 mm and GTV - 4 mm were generated only for GTVs of ≥0.72 cc and ≥2.20 cc, respectively, to ensure the minimum meaningful volume for evaluation [16,17].

An irradiated isodose volume (IIV) was defined as the total volume irradiated with more than a certain relevant dose, including the GTV [2,27]. The IIVs of 100, 75, and 50% of the GTV *D*_V-0.01 cc_ (100, 75, and 50% of PIV, prescribed isodose volume) were calculated from the dose-volume histogram (DVH) for the volume generated by adding an isotropic 10-30 mm margin to each GTV boundary [16]. The absolute volumes obtained by subtracting the GTV from these IIVs were recorded as each spillage volume. The GTV near-maximum dose (*D*_near-max_) was recorded as *D*_0.01 cc_, the minimum dose covering 0.01 cc of the GTV, for GTV ≥0.20 cc or *D*_5%_ (*D*_<0.01 cc_) for GTV <0.20 cc [14,16]. The GTV dose inhomogeneity was defined as the GTV *D*_V-0.01 cc_ (%) relative to the GTV *D*_near-max_ (100%) as defined above [16]. The near-minimum doses of GTV, GTV + 2 mm, GTV - 2 mm, and GTV - 4 mm structures were evaluated as each *D*_eIIV_ (eIIV: equivalent IIV), the minimum dose to cover the IIV equivalent to each target volume (TV) on the DVH [15,17]. Each *D*_eIIV_ was recorded as the relative % dose to the GTV *D*_V-0.01 cc_ (100%) [15,17]. The coverage value (%) of each reference TV by the *D*_eIIV_ was also recorded. While a high value of GTV *D*_eIIV_ is an indicator of the steepness of the dose increase just inside the prescribed isodose surface [17], the closer the GTV *D*_eIIV_ is to the prescribed dose (GTV *D*_V-0.01 cc_), the better the dose conformity is [16]. The GTV dose conformity was compared using the smallness of the PIV spillage (cc) outside the GTV, the high GTV coverage value (%) by the *D*_eIIV_, and the closeness of GTV *D*_eIIV_ to the GTV *D*_V-0.01 cc_. The appropriateness of the dose attenuation margin outside the GTV was compared using the GTV + 2 mm *D*_eIIV_ relative to the GTV *D*_V-0.01 cc_ and the GTV coverage value by the *D*_eIIV_ [15]. The steepness of dose gradients outside the GTV and GTV + 2 mm were compared using the smallness of 75 and 50% PIV spillage volumes [14,16]. The steepness of dose increase inside the GTV boundary was compared using the *D*_eIIV_s (%) of the GTV, GTV - 2 mm, and GTV - 4 mm [17]. The concentric lamellarity of dose gradients outside and inside the GTV boundary was compared using the high coverage rates of GTV + 2 mm, GTV - 2 mm, and GTV - 4 mm by each *D*_eIIV_ [15,17].

For statistical analyses, paired nonparametric tests were adopted, considering the dominant distributions of variables. Box-and-whisker plots (BWPs) were used to show the distribution of variables. In the BWP, the whiskers indicate the nearest values ≤1.5 times the IQR. The cross marks beyond the lines denote the outliers >1.5 times the IQR. Friedman’s test (FT) and Scheffe’s post hoc test (SPHT) were used to compare four numerical variables. The Wilcoxon signed-rank test (WSRT) was used to compare the two numerical variables. If there was no significant difference between the two numerical variables in the SPHT and the p-value was <0.9, WSRT was additionally applied to compare them. The Jonckheere-Terpstra (JT) test was used to assess a trend of increase or decrease in dosimetric parameters with the reduction of GS and SU between four variables [10]. Statistical significance was considered at p < 0.05 (*), p < 0.01 (**) and p < 0.001 (***). Statistical analyses were performed using BellCurve for Excel (version 4.05; Social Survey Research Information Co., Ltd., Tokyo, Japan).

## Results

**Figure 1:**
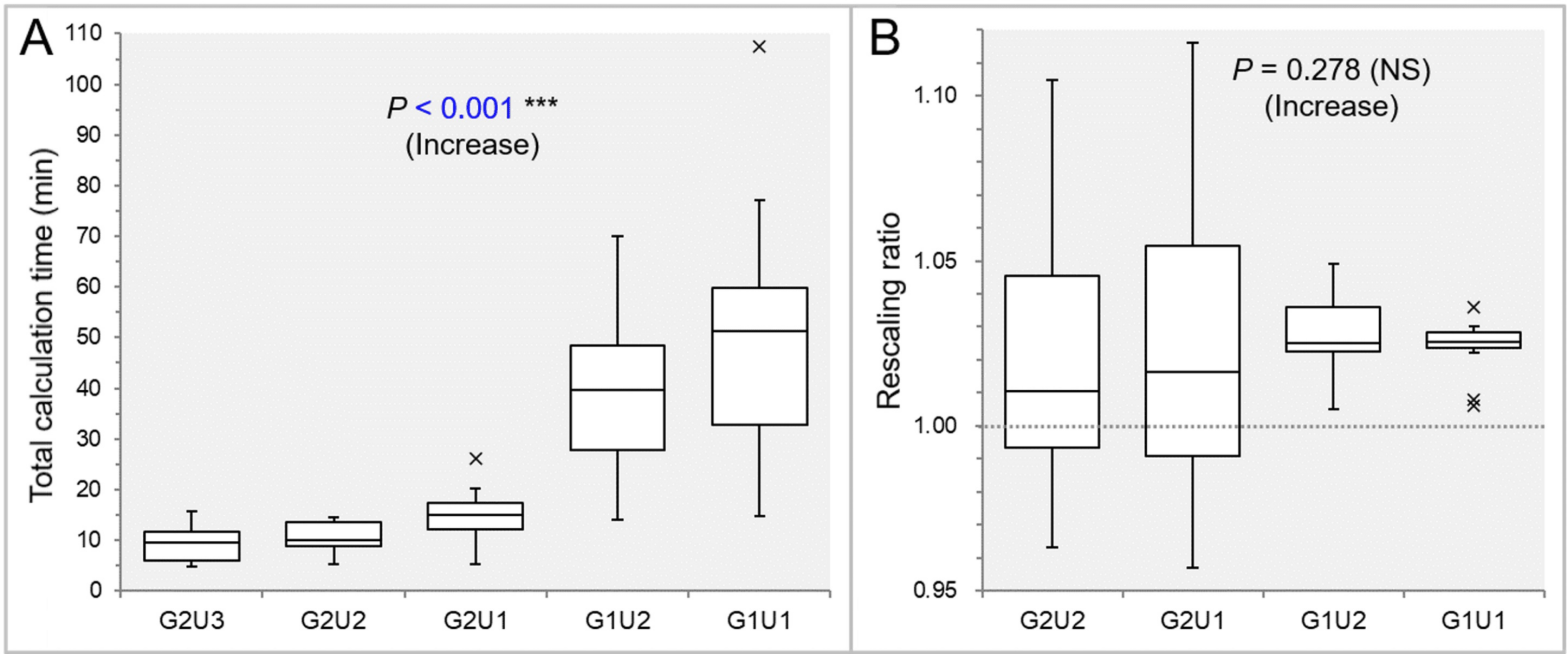
Comparison of total calculation time and rescaling ratio to align the prescription dose after optimization. The images show box-and-whisker plots (BWPs) (A,B) for comparisons of the total calculation time (tCT) (A) and rescaling ratio (B) between the five (A) and four (B) groups, respectively. The results of the Jonckheere-Terpstra (JT) test are attached along with trends (A,B). The dotted line in B shows the 1.000 value meaning no need for rescaling. GXUY: grid spacing (G) is X mm and statistical uncertainty (U) is Y%.

The dosimetric parameters of the G2U3 and G2U2 were exactly the same in the 12 GTVs. There was no significant difference in the tCT between the G2U3 and G2U2 (WSRT, p = 0.981) (Figure 1A).

The results of the planning and dosimetric comparisons between the four groups for the 12 GTVs are shown in Tables 3, 4.

**Table 3:** Comparison between the four different combinations of grid spacing and statistical uncertainties: Part 1. If a p-value of Scheffe’s post hoc test (SPHT) is <0.9, the result of Wilcoxon signed-rank test (WSRT) is attached. GXUY: grid spacing (G) is X mm and statistical uncertainty (SU) is Y%; vs: versus; tCT: total calculation time; PIV: prescribed isodose volume; GTV: gross tumor volume; *D*_eIIV_: the minimum dose to cover the irradiated isodose volume equivalent to a target volume; GTV + 2 mm: GTV evenly expanded by 2 mm; X% PIV: the volume irradiated with ≥X% of the prescribed dose, including a target volume; NS: not significant.

Parameter		tCT (min)	Rescaling ratio	PIV spillage (cc)	GTV *D*_eIIV_ coverage (%)	GTV + 2 mm *D*_eIIV_ (%)	GTV + 2 mm *D*_eIIV_ coverage (%)	75% PIV spillage (cc)	50% PIV spillage (cc)
Friedman’s test (p-value)		< 0.001 ***	0.988 (NS)	< 0.001 ***	< 0.001 ***	< 0.001 ***	0.072 (NS)	< 0.001 ***	< 0.001 ***
Scheffe’s post hoc test [Wilcoxon signed-rank test]: p-value	G2U2 vs G2U1	0.475 (NS) [0.003**]	0.996 (NS)	0.940 (NS)	0.514 (NS) [0.033 *]	0.984 (NS)	0.497 (NS) [0.011 *]	0.9999 (NS)	0.976 (NS)
	G2U2 vs G1U2	< 0.001 ***	0.9999 (NS)	0.001 **	0.014 *	0.011 *	0.944 (NS)	0.004 **	0.037 (NS) [0.016 *]
	G2U2 vs G1U1	< 0.001 ***	0.992 (NS)	0.004 **	< 0.001 ***	0.022 *	0.107 (NS) [0.041 *]	0.005 **	0.010 *
	G2U1 vs G1U2	0.094 (NS) [0.002 **]	0.999 (NS)	0.012 *	0.380 (NS) [0.041 *]	0.003 **	0.836 (NS) [0.530 (NS)]	0.005 **	0.010 *
	G2U1 vs G1U1	0.007 **	0.9999 (NS)	0.029 *	0.074 (NS) [0.023 *]	0.007 **	0.836 (NS) [0.328 (NS)]	0.007 **	0.002 **
	G1U2 vs G1U1	0.825 [0.060 (NS)]	0.996 (NS)	0.992 (NS)	0.857 (NS) [0.158 (NS)]	0.997 (NS)	0.330 (NS) [0.091 (NS)]	0.9999 (NS)	0.976 (NS)

**Table 4:** Dosimetric comparison between the four different combinations of grid spacing and statistical uncertainties: Part 2. If a p-value of Scheffe’s post hoc test (SPHT) is <0.9, the result of Wilcoxon signed-rank test (WSRT) is attached. GXUY: grid spacing (G) is X mm and statistical uncertainty (SU) is Y%; vs: versus; GTV: gross tumor volume; *D*_V-0.01 cc_: the minimum dose to cover a target volume minus 0.01 cc; IDS: isodose surface; *D*_eIIV_: the minimum dose to cover the irradiated isodose volume equivalent to a target volume; GTV – X mm: GTV evenly reduced by X mm; NS: not significant.

Parameters		GTV *D*_V-0.01 cc_ % IDS (%)	GTV *D*_eIIV_ (%)	GTV – 2 mm *D*_eIIV_ (%)	GTV – 2 mm *D*_eIIV_ coverage (%)	GTV – 4 mm *D*_eIIV_ (%)	GTV – 4 mm *D*_eIIV_ coverage (%)
Friedman’s test (p-value)		0.377 (NS)	< 0.001 ***	0.960 (NS)	< 0.001 ***	1.0000 (NS)	< 0.001 ***
Scheffe’s post hoc test [Wilcoxon signed-rank test]: p-value	G2U2 vs G2U1	0.567 (NS) [0.158 (NS)]	0.514 (NS) [0.433 (NS)]	0.982 (NS)	0.842 (NS) [0.142 (NS)]	1.0000 (NS)	0.861 (NS) [0.508 (NS)]
	G2U2 vs G1U2	0.999 (NS)	0.014 *	0.970 (NS)	0.047 *	1.0000 (NS)	0.034 *
	G2U2 vs G1U1	0.940 (NS)	<0.001 ***	0.982 (NS)	<0.001 ***	1.0000 (NS)	0.002 **
	G2U1 vs G1U2	0.475 (NS) [0.239 (NS)]	0.380 (NS) [0.003 **]	0.9998 (NS)	0.303 (NS) [0.051 (NS)]	1.0000 (NS)	0.229 (NS) [0.047 *]
	G2U1 vs G1U1	0.891 (NS) [0.433 (NS)]	0.074 (NS) [0.005 **]	1.0000 (NS)	0.012 *	1.0000 (NS)	0.034 *
	G1U2 vs G1U1	0.891 (NS) [0.239 (NS)]	0.857 (NS) [0.480 (NS)]	0.9998 (NS)	0.575 (NS) [0.0502 (NS)]	1.0000 (NS)	0.861 (NS) [0.037 *]

There were significant differences in FT, except for the rescaling ratio, the GTV + 2 mm coverage by *D*_eIIV_, the GTV dose inhomogeneity, and the *D*_eIIV_s of GTV - 2 mm and GTV - 4 mm.

The tCT increased significantly as the GS and SU became smaller (Table 3 and Figure 1A). The tCT was significantly longer in the G1U1 and G1U2 than in the G2U1 and G2U2, while there was no significant difference between the G1U2 and G1U1 (Table 3 and Figure 1A). The variations in the rescaling ratios were remarkable in the G2U2 and G2U1, compared to the G1U2 and G1U1 (Table 3 and Figure 1B). The rescaling ratio in the G1U1 had the least variation (Figure 1B).

**Figure 2:**
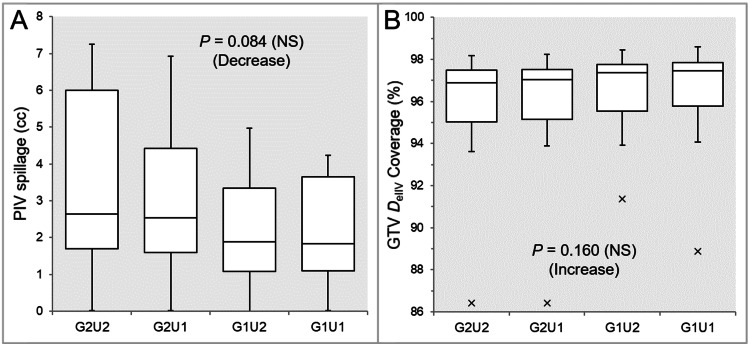
Comparison of GTV dose conformity between the four groups. The images show BWPs (A,B), along with the results of the JT test, for comparisons of the PIV spillage volume outside the GTV boundary (A) and the GTV coverage value by the *D*_eIIV_ (B) between the four groups. GTV: gross tumor volume; GXUY: grid spacing (G) is X mm and statistical uncertainty (SU) is Y%; PIV: prescribed isodose volume; *D*_eIIV_: the minimum dose to cover the irradiated isodose volume equivalent to a target volume; NS: not significant; BWPs: box-and-whisker plots; JT: Jonckheere-Terpstra.

The PIV spillage was smaller in the G1U2 and G1U1 than in the G2U1 and G2U2, while there was no significant difference between the G1U2 and G1U1 (Table 3 and Figure 2A).

The GTV coverage values by the DeIIV were higher in the G1U2 and G1U1 than in the G2U1 and G2U2, while there was no significant difference between the G1U2 and G1U1 (Table 3 and Figure 2B).

**Figure 3:**
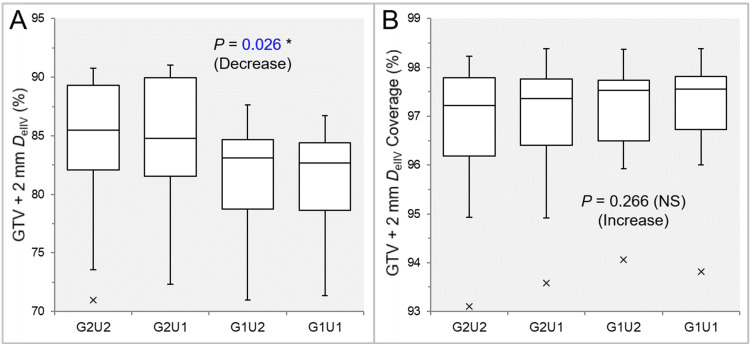
Comparison of the appropriateness of dose attenuation margin outside the GTV between the four groups. The images show BWPs (A,B), along with the results of the JT test, for comparisons of the GTV + 2 mm *D*_eIIV_ (%) relative to the GTV *D*_V-0.01 cc_ (100%) (A) and the coverage value of GTV + 2 mm by the *D*_eIIV_ (B) between the four groups. GTV: gross tumor volume; GXUY: grid spacing (G) is X mm and statistical uncertainty (SU) is Y%; GTV + 2 mm: GTV evenly expanded by 2 mm; *D*_eIIV_: the minimum dose to cover the irradiated isodose volume equivalent to a target volume; NS: not significant; BWPs: box-and-whisker plots; JT: Jonckheere-Terpstra; *D*_V-0.01 cc_: the minimum dose to cover a target volume minus 0.01 cc.

There was a significant trend of decrease in the GTV + 2 mm *D*_eIIV_ (Figure 3A).

The GTV + 2 mm *D*_eIIV_s were significantly lower and more appropriate in the G1U2 and G1U1 than in the G2U1 and G2U2, while there was no significant difference between the G1U2 and G1U1 (Table 3 and Figure 3A). The coverage value of GTV + 2 mm by the *D*_eIIV_ was significantly lower in the G2U2 than in the G2U1 and G1U1, while there was no significant difference between the other groups (Table 3 and Figure 3B).

**Figure 4:**
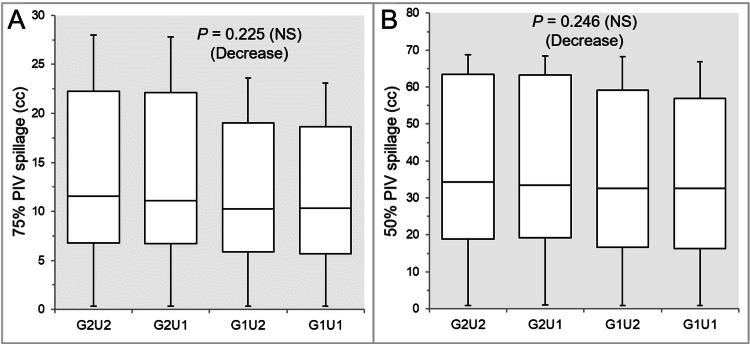
Comparisons of the steepness of the dose gradient outside the GTV between the four groups. The images show BWPs (A,B), along with the results of the JT test, for comparisons of the 75% (A) and 50% (B) PIV spillage volumes outside the GTV between the four groups. GTV: gross tumor volume; GXUY: grid spacing (G) is X mm and statistical uncertainty (SU) is Y%; PIV: prescribed isodose volume; X% PIV: the isodose volume irradiated with ≥X% of the prescribed dose (GTV *D*_V-0.01 cc_), including a target volume; *D*_V-0.01 cc_: the minimum dose to cover a target volume minus 0.01 cc; NS: not significant; BWPs: box-and-whisker plots; JT: Jonckheere-Terpstra.

The 75 and 50% PIV spillages were significantly smaller in the G1U2 and G1U1 than in the G2U1 and G2U2, while there were no significant differences between the G1U2 and G1U1 (Table 3 and Figures 4A, 4B).

**Figure 5:**
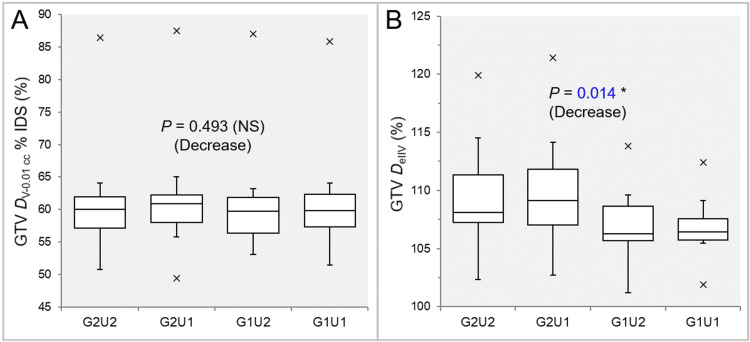
Comparisons of the GTV dose inhomogeneity and the steepness of dose increase just inside the prescribed isodose surface between the four groups. The images show BWPs (A,B), along with the results of the JT test, for comparisons of the GTV *D*_V-0.01 cc_ (%) relative to the *D*_0.01 cc_ (100%) (A) and the GTV *D*_eIIV_ (%) relative to the *D*_V-0.01 cc_ (100%) (B) between the four groups. GTV: gross tumor volume; GXUY: grid spacing (G) is X mm and statistical uncertainty (SU) is Y%; *D*_V-0.01 cc_: the minimum dose to cover a target volume minus 0.01 cc; IDS: isodose surface; *D*_eIIV_: the minimum dose to cover the irradiated isodose volume equivalent to a target volume; NS: not significant; BWPs: box-and-whisker plots; JT: Jonckheere-Terpstra; *D*_0.01 cc_: the minimum dose covering 0.01 cc of a target volume.

There was no significant difference in the GTV dose inhomogeneity (Table 4 and Figure 5A).

There was a significant trend of decrease in the GTV *D*_eIIV_ (Figure 5A). The GTV *D*_eIIV_s were significantly lower and closer to the prescribed dose (100%) in the G1U2 and G1U1 than in the G2U1 and G2U2, while there was no significant difference between the G1U2 and G1U1 (Table 4 and Figure 5B).

**Figure 6:**
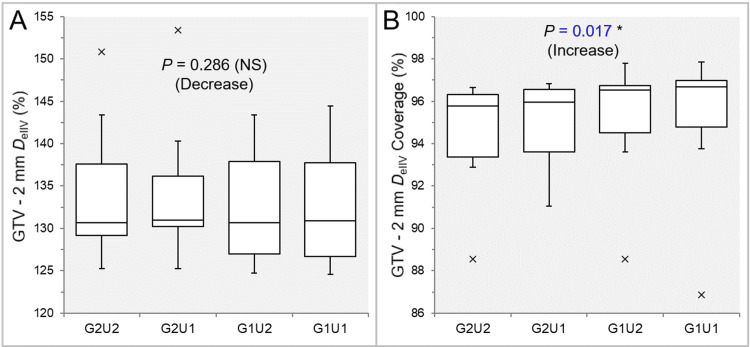
Comparison of the steepness of dose increase and the concentric lamellarity at 2 mm inside the GTV boundary between the four groups. The images show BWPs (A,B), along with the results of the JT test, for comparisons of the GTV - 2 mm *D*_eIIV_ (%) relative to the GTV *D*_V-0.01 cc_ (100%) (A) and the coverage value of GTV - 2 mm by the *D*_eIIV_ (B) between the four groups. GTV: gross tumor volume; GXUY: grid spacing (G) is X mm and statistical uncertainty (SU) is Y%; GTV – 2 mm: GTV evenly reduced by 2 mm; *D*_eIIV_: the minimum dose to cover the irradiated isodose volume equivalent to a target volume; NS: not significant; BWPs: box-and-whisker plots; JT: Jonckheere-Terpstra; *D*_V-0.01 cc_: the minimum dose to cover a target volume minus 0.01 cc.

There was no significant difference in the GTV - 2 mm *D*_eIIV_s in the 11 GTVs (Table 4 and Figure 6A).

There was a significant trend of increase in the coverage value of GTV - 2 mm by the *D*_eIIV_ (Figure 6B). The coverage values of GTV - 2 mm by the *D*_eIIV_ were significantly higher in the G1U2 and G1U1 than in the G2U1 and G2U2, while there was no significant difference between the G1U2 and G1U1 (Table 4 and Figure 6B).

**Figure 7:**
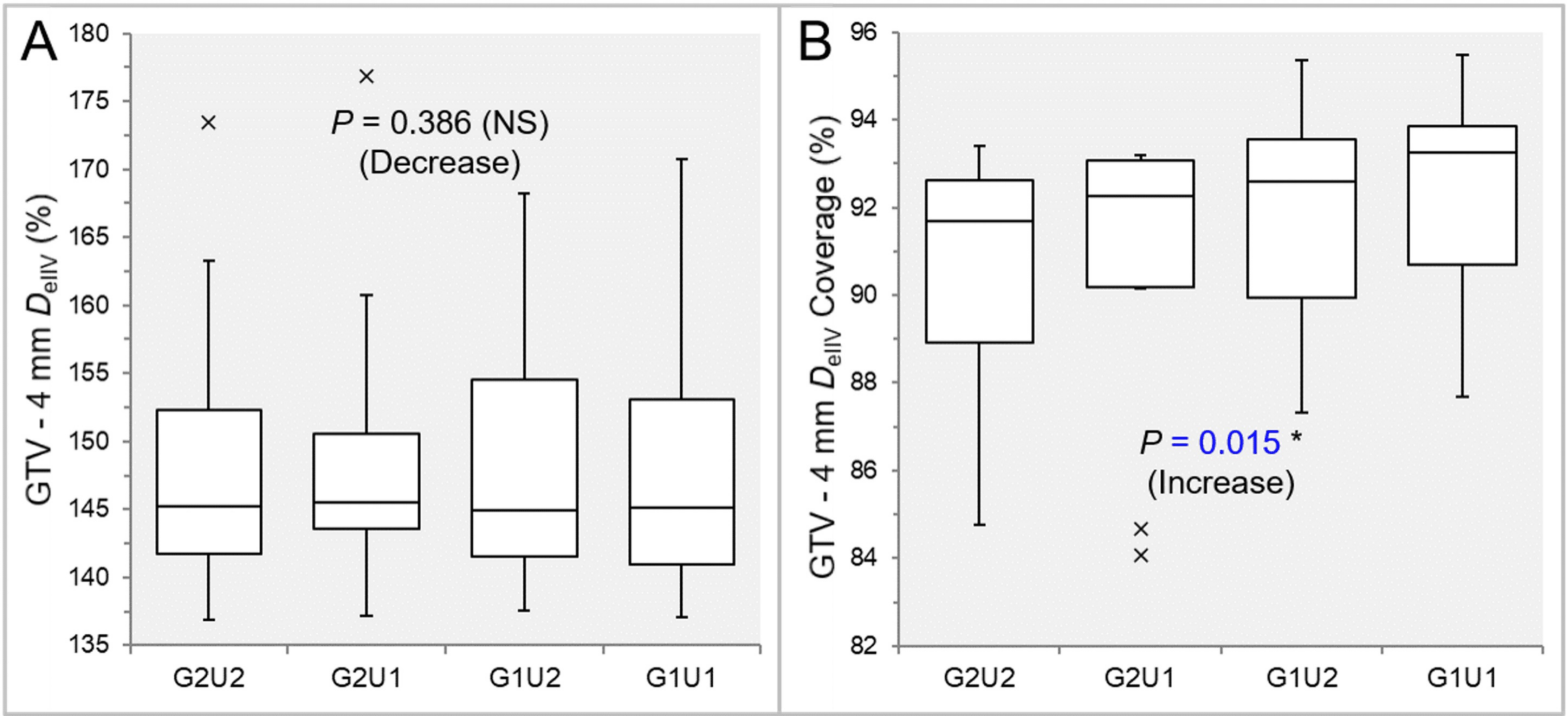
Comparison of the steepness of dose increase and the concentric lamellarity at 4 mm inside the GTV between the four groups. The images show BWPs (A,B), along with the results of JT test, for comparisons of the GTV - 4 mm *D*_eIIV_ (%) relative to the GTV *D*_V-0.01 cc_ (100%) (A) and the coverage value of GTV - 4 mm by the *D*_eIIV_ (B) between the four groups. GTV: gross tumor volume; GXUY: grid spacing (G) is X mm and statistical uncertainty (SU) is Y%; GTV – 4 mm: GTV evenly reduced by 4 mm; *D*_eIIV_: the minimum dose to cover the irradiated isodose volume equivalent to a target volume on the dose-volume histogram; NS: not significant; BWPs: box-and-whisker plots; JT: Jonckheere-Terpstra; *D*_V-0.01 cc_: the minimum dose to cover a target volume minus 0.01 cc.

There was no significant difference in the GTV - 4 mm *D*_eIIV_s in the 10 GTVs (Table 4 and Figure 7A).

There was a significant trend of increase in the coverage value of GTV - 4 mm by the *D*_eIIV_ (Figure 7B). The coverage values of GTV - 4 mm by the *D*_eIIV_ were significantly higher in the G1U2 and G1U1 than in the G2U1 and G2U2, while there was no significant difference between the G1U2 and G1U1 in SPHT (Table 4 and Figure 7B). However, the GTV - 4 mm coverage by the *D*_eIIV_ was significantly higher in the G1U1 than in the G1U2 in WSRT (Table 4 and Figure 7B).

To summarize the results so far, the dosimetric parameters were significantly superior in the G1U2 and G1U1 than in the G2U1 and G2U2, while there were no significant differences between the G1U2 and G1U1, except for the GTV - 4 mm coverage by the *D*_eIIV_. Therefore, the differences in planning and dosimetric parameters between the G1U2 and G1U1 were compared by WSRT using the 30 GTVs.

**Figure 8:**
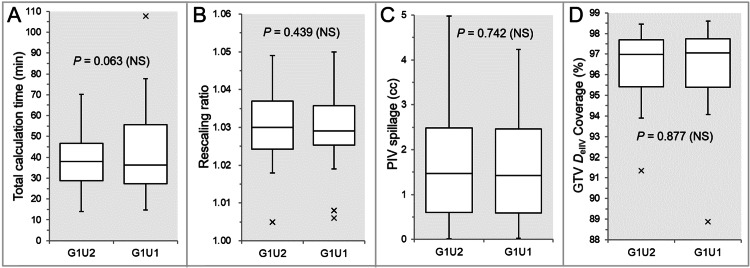
Comparisons of total calculation time, rescaling ratio, and the GTV dose conformity between the two groups, G1U2 and G1U1. The images show BWPs (A-D), along with the results of WSRT, for comparisons of the total calculation time (A), rescaling ratio (B), the PIV spillage volume outside the GTV boundary (C), and the GTV coverage value by the *D*_eIIV_ (D) between the two groups. GTV: gross tumor volume; GXUY: grid spacing (G) is X mm and statistical uncertainty (SU) is Y%; PIV: prescribed isodose volume; *D*_eIIV_: the minimum dose to cover the irradiated isodose volume equivalent to a target volume; NS: not significant; BWPs: box-and-whisker plots; WSRT: Wilcoxon signed-rank test.

**Figure 9:**
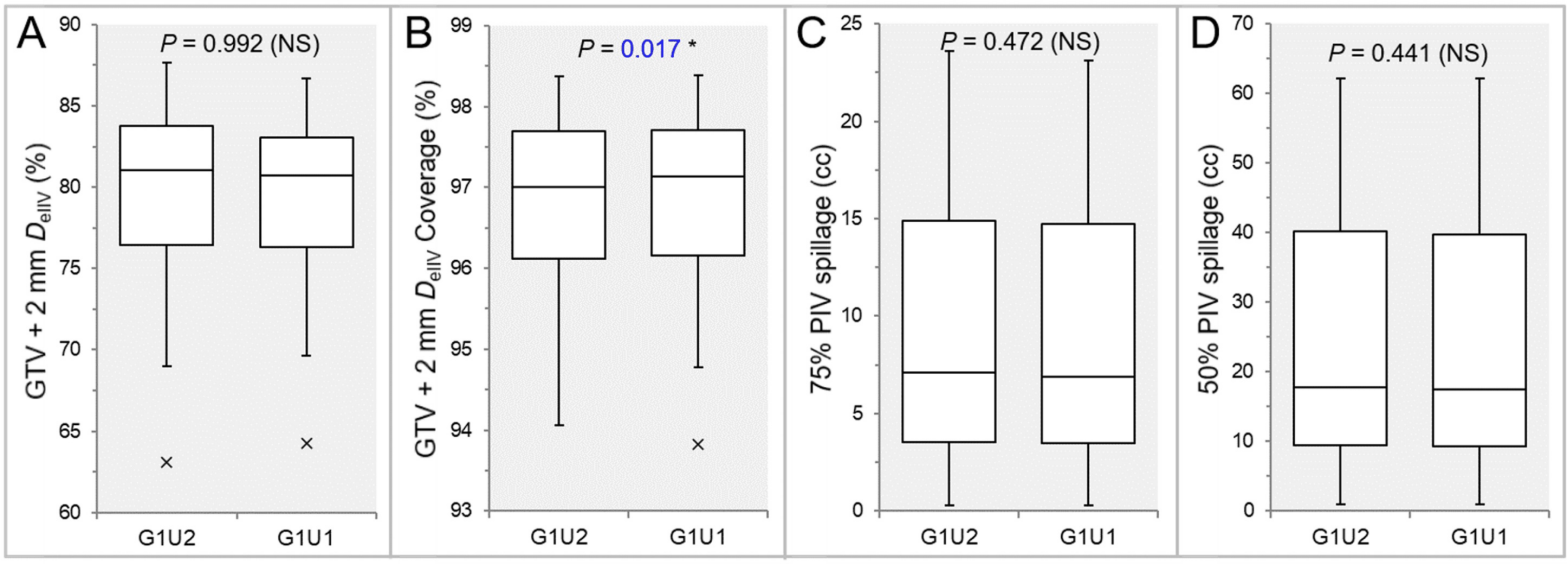
Comparisons of the appropriateness of dose attenuation margin outside the GTV and the steepness of dose gradient outside the GTV between the two groups. The images show BWPs (A-D), along with the results of WSRT, for comparisons of the total calculation time (A), rescaling ratio (B), the PIV spillage volume outside the GTV boundary (C), and the GTV coverage value by the *D*_eIIV_ (D) between the two groups. GTV: gross tumor volume; GXUY: grid spacing (G) is X mm and statistical uncertainty (SU) is Y%; GTV + 2 mm: GTV evenly expanded by 2 mm; *D*_eIIV_: the minimum dose to cover the irradiated isodose volume equivalent to a target volume on the dose-volume histogram; PIV: prescribed isodose volume; X% PIV: the isodose volume irradiated with ≥X% of the prescribed dose (GTV *D*_V-0.01 cc_), including a target volume; GTV: gross tumor volume; *D*_V-0.01 cc_: the minimum dose to cover a target volume minus 0.01 cc; NS: not significant; BWPs: box-and-whisker plots; WSRT: Wilcoxon signed-rank test.

**Figure 10:**
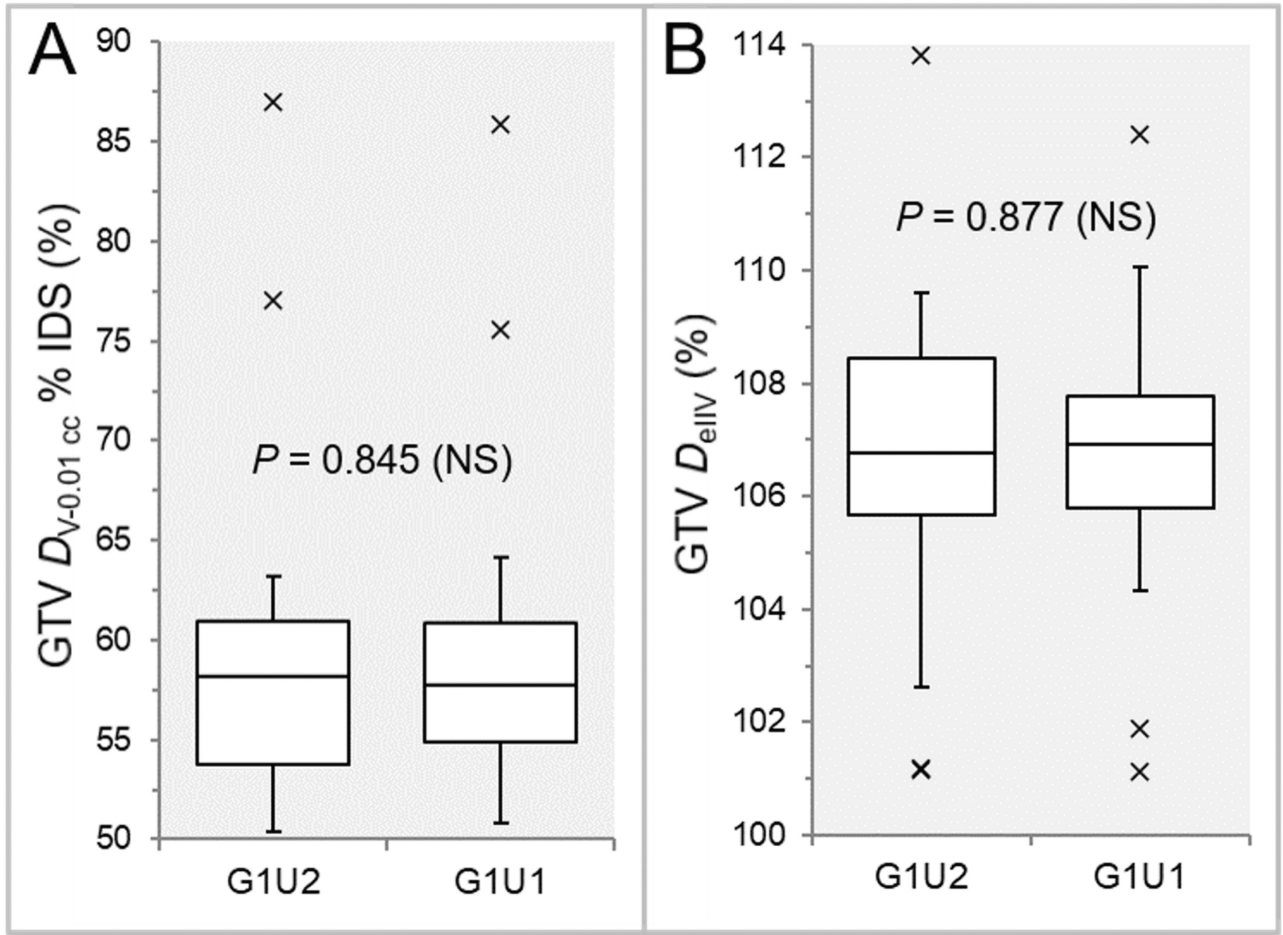
Comparisons of the GTV dose inhomogeneity and the steepness of dose increase just inside the prescribed isodose surface between the two groups. The images show BWPs (A,B), along with the results of WSRT, for comparisons of the GTV *D*_V-0.01 cc_ (%) relative to the *D*_0.01 cc_ (100%) (A) and the GTV *D*_eIIV_ (%) relative to the *D*_V-0.01 cc_ (100%) (B) between the four groups. GTV: gross tumor volume; GXUY: grid spacing (G) is X mm and statistical uncertainty (SU) is Y%; *D*_V-0.01 cc_: the minimum dose to cover a target volume minus 0.01 cc; IDS: isodose surface; *D*_eIIV_: the minimum dose to cover the irradiated isodose volume equivalent to a target volume; NS: not significant; BWPs: box-and-whisker plots; WSRT: Wilcoxon signed-rank test; *D*_0.01 cc_: the minimum dose covering 0.01 cc of a target volume.

**Figure 11:**
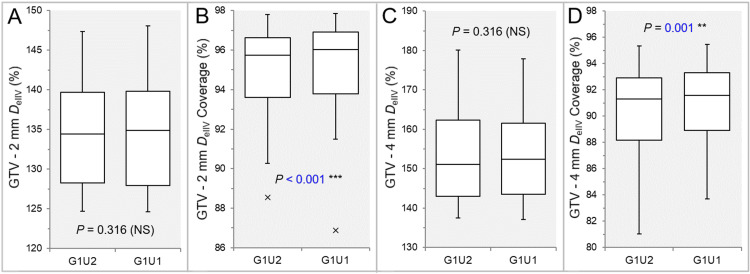
Comparison of the steepness of dose increase 2-4 mm inside the GTV and the concentric lamellarity between the two groups. The images show BWPs (A-D), along with the results of WSRT, for comparisons of the *D*_eIIV_ (%) of GTV – 2 mm (A) and GTV - 4 mm (C) relative to the GTV *D*_V-0.01 cc_ (100%) and each coverage value by the *D*_eIIV_ (B,D) between the two groups. GTV: gross tumor volume; GXUY: grid spacing (G) is X mm and statistical uncertainty (SU) is Y%; GTV – X mm: GTV evenly reduced by X mm; *D*_eIIV_: the minimum dose to cover the irradiated isodose volume equivalent to a target volume; NS: not significant; BWPs: box-and-whisker plots; WSRT: Wilcoxon signed-rank test; *D*_V-0.01 cc_: the minimum dose to cover a target volume minus 0.01 cc.

There were no significant differences between the G1U2 and G1U1 (Figures 8A, 8B, 8C, 8D, 9A, 9C, 9D, 10A, 10B, 11A, 11C), except for the coverage values of GTV + 2 mm, GTV - 2 mm (28 GTVs), and GTV - 4 mm (26 GTVs) by each *D*_eIIV_ (Figures 9B, 11B, 11D).

The coverage values of GTV + 2 mm, GTV - 2 mm, and GTV - 4 mm by each *D*_eIIV_ were significantly higher in the G1U1 than in the G1U2 (Figures 9B, 11B, 11D).

The GTV dose conformity and the steepness of the dose gradient outside the GTV boundary tended to be superior in the G1U1 than in the G1U2, although there were no statistically significant differences (Figures 8C, 8D, 9A, 9C, 9D). However, the tCT tended to be longer in the G1U1 than in the G1U2, especially for large GTVs (Figure 8A).

## Discussion

This study revealed the initial settings of calculation GS and SU for the XVMC algorithm have significant impacts on the plan quality. First, it was interesting that the dose distributions of the G2U3 and G2U2 were exactly the same; that is, the optimization results for SU per plan between 3% and 2% were equivalent, although the tCTs were different. Furthermore, the difference in the initial setting of the calculation grid size, GS of 2 mm or 1 mm, significantly affected the optimization results. In the SU of 1 mm, the tCT was significantly longer; however, the dispersion and quality of dose distribution were significantly improved in terms of the GTV dose conformity, the steepness of dose falloff outside the GTV, and the degree of concentric lamellarity of dose gradients outside and inside the GTV boundary. These differences in the plan quality can lead to a difference in the degree of adverse radiation effect, especially for large and/or irregular lesions, if the same dose is prescribed to the GTV margin (GTV DV-0.01 cc). Meanwhile, the only significant difference between the G1U2 and G1U1, i.e., SU of 2% and 1%, was that the SU of 1% was superior in the concentric lamellarity of dose gradients just outside and inside the GTV boundary. This difference may affect the control of microscopic brain invasion and tumor response early after the initiation of irradiation [15,17]. Superior dose distribution should be prioritized over shorter tCT to improve long-term treatment outcomes. Based on these results, the initial settings for VMA-based SRS of single or oligo BMs were changed to GS of 1 mm and SU per plan of 1% at our facility.

In addition, the initial settings of GS and SU for all VMA therapy other than SRS for BMs were changed to the SU of 1% per plan and the GS of 2 or 3 mm, resulting in improved dose distribution in most cases. The obvious disadvantage of making the GS and SU smaller is that the tCT increases. However, the adoption of GPU (graphics processing unit) is expected to significantly reduce the tCT compared to the current CPU (central processing unit)-based version.

In this study, dosimetric comparisons were made using highly unique methods different from the general evaluation metrics [28,29]. We have previously revealed the issues inherent in the evaluation methods using indicators such as ICRU recommendations [14,30], conformity index [28], and gradient index [29], and advocated alternatives [14,15,17]. These include dose metrics of the GTV boundary and 2 mm inside and outside it. In the comparison between the G1U2 and G1U1, the coverage values of GTV + 2 mm, GTV - 2 mm, and GTV - 4 mm by each *D*_eIIV_ were the only significant differences. The degree of concentric lamellarity at the GTV boundary and the dose gradients outside and inside it reflect a high degree of overall dose conformity.

Study limitations

This study did not examine the SU of <1% or 1.5% per plan and an SU per control point [18-20]. Therefore, we cannot conclude that the GS of 1 mm and SU of 1% are optimal for VMA-based SRS using Monaco®. In addition, the total monitor units per fraction and total irradiation time were not compared. Improving the quality of the treatment plan tends to increase the irradiation time, so it is necessary to verify whether it is within an acceptable range. This study was also limited to single BMs. One of the true values of VMA is the simultaneous irradiation of multiple BMs with a single isocenter. The optimal GS and SU settings for multiple lesions of 5-10 or more remain to be examined. In the settings of GS of 1 mm and SU of 1%, optimization may not progress smoothly if irradiating 10-20 or more lesions simultaneously. This study was a comparison limited to treatment planning and should be compared comprehensively, including differences in the patient’s specific quality assurance, delivery time, and clinical outcomes.

## Conclusions

In VMA-based SRS for BMs using Monaco^®^, the initial settings of calculation GS and SU for the XVMC algorithm have significant impacts on the plan quality and tCT. The settings with GS of 1 mm and SU of 1% per plan are recommended to create the most suitable dose distribution for single BMs, especially for irregularly shaped and/or large lesions, although the tCT is long. In addition to common evaluation metrics, the coverage values of 2 mm outside and 2-4 mm inside the GTV surface by the *D*_eIIV_ are valuable for in-depth plan comparison. 
